# The willingness of Saudi men with type 2 diabetes to discuss erectile dysfunction with their physicians and the factors that influence this

**DOI:** 10.1371/journal.pone.0201105

**Published:** 2018-07-25

**Authors:** Turky H. Almigbal, Peter Schattner

**Affiliations:** 1 Department of Family and Community Medicine, College of Medicine, King Saud University, Riyadh, Saudi Arabia; 2 Department of General Practice, School of Primary Health Care, Faculty of Medicine, Nursing and Health Sciences, Monash University, Melbourne, Australia; The University of Warwick, UNITED KINGDOM

## Abstract

**Objectives:**

The study’s objectives were to find out the proportion of Saudi men with type 2 diabetes who have been asked by their physicians about erectile dysfunction (ED) in the last year, to determine the willingness of Saudi men with type 2 diabetes to discuss ED, and to explore the factors that may be related to their willingness to discuss ED with their physicians.

**Methods:**

This study employed a cross-sectional survey design using a quantitative self-administered questionnaire among 309 Saudi men with type 2 diabetes. The study was conducted in hospital-based primary care clinics at King Khalid University Hospital, Riyadh, Saudi Arabia during the period from July to September 2015.

**Results:**

The mean age of the patients was 60.2 years with the mean duration of diabetes approximately 12.5 years. Few of the patients (9.7%) had been asked by their physicians about ED within the last year of attending the clinics although most patients (84.8%) were willing to discuss this issue. The presence of ED among the respondents was 89%. Two participants’ characteristics were associated with a willingness to discuss ED with the physicians. These characteristics were age above 60 (OR = 0.25, 95% CI: 0.11–0.55), and having severe ED (OR = 0.26, 95% CI: 0.08–0.85). The respondents’ main barriers to discussing ED with their physicians were embarrassing the doctor, ED is a personal issue, too old or too sick to address ED issues now, no effective treatment available, and the doctor is too young to discuss ED with.

**Conclusions:**

Most patients who have type 2 diabetes are not asked about ED within the last year of attendance even though most are willing to discuss it with their physicians. Being older and suffering more severe ED will result in being less willing to discuss ED with their doctor. Further research is needed to explore the barriers which prevent physicians from discussing ED with their patients who have diabetes.

## Introduction

Diabetes mellitus is one of the most common chronic diseases worldwide, with an increasing incidence in most countries.[1] There were more than 382 million people with diabetes mellitus in 2013 and this is forecasted to reach 592 million people by 2035.[2–3] Most of the deaths among patients with diabetes are not due to diabetes itself but due to the complications associated with it. This includes cardiovascular disease which can cause about 50% of the deaths among patients with diabetes.[2] In Saudi Arabia, diabetes mellitus has become an overwhelming health problem with an overall prevalence among adults of approximately 23.7%.[4] The complications that are usually associated with diabetes mellitus are due to macrovascular or microvascular disease.[5–7] The prevalence of these complications in Saudi Arabia is quite high. In one study done in that country, the prevalence of complications were: myocardial infarction (14.3%), retinopathy (16.7%), acute coronary syndrome (23.1%) and nephropathy (32.1%).[8]

Erectile dysfunction (ED) is a common association of diabetes and is caused by a neuropathy or vasculopathy.[9–10] ED is defined as “the persistent inability to attain and maintain an erection that is sufficient to permit satisfactory sexual performance”.[11] There is some evidence that suggests that low testosterone might be involved in both the development of type 2 diabetes and the subsequent complication of ED. [12] Several epidemiological studies have shown that both type 1 and type 2 diabetes are associated with higher risks of ED.[13] Also, it has been recognized that ED can be found even in preclinical or newly diagnosed diabetes. [10, 14–16] The prevalence of ED among men with diabetes ranges from 35% to 90% depending on the method used to identify it.[17] In Saudi Arabia, studies have shown that the overall prevalence of ED in men with diabetes range from 63.5% to 83%.[18–19] There is a threefold increased risk of ED in men with diabetes compared to men without diabetes.[20] Furthermore, even after adjusting the risk of ED for age in men with diabetes, the risk is still double compared to those without the disease.[21]

Moreover, ED in men with diabetes occurs 10–15 years earlier than in men without diabetes. [20] It has been shown that quality of life is reduced in men with diabetes who are suffering from ED.[22] In addition, ED is considered a predictor for cardiovascular events and can be associated with silent myocardial ischemia among men with type 2 diabetes mellitus.[23–24] In addition to that, ED in people with diabetes can be the first sign of future cardiovascular events. [24] The existence of ED in men with diabetes is a reason to screen for other diabetic complications caused by microangiopathy in target organs.[25]

Doctors can diagnose ED by several methods. A detailed medical history and physical examination can give a good idea about its causes or degree of severity.[26] Obviously, the most important step to start with is to simply ask men with diabetes about this problem during a routine clinical review. The UK NICE guidelines for diabetes mellitus recommend that “Review the issue of erectile dysfunction with men annually”.[27]

Surprisingly, few doctors ask men with diabetes about ED and this problem is frequently overlooked. For example, in one study done in England only 9% of men with diabetes were asked by their physicians about ED.[26] In another study done in the United states, physicians initiated the discussion about ED with only 18% of their patients with diabetes.[28] On the other hand, very few studies have addressed the issue of the discussion of ED from the perspective of the patient with diabetes, for example, whether they are willing to be asked about ED by their physicians. For example, a study done in Taiwan showed that 56.6% of patients with diabetes wished to discuss ED with their physicians.[29]

The literature indicates that barriers that might prevent health professionals from asking about sexual problems such as ED include lack of time or knowledge, lack of training among physicians, false beliefs about sexuality, thinking that this is a job for another physician, patients not being ready to discuss these issues, believing it is not an important subject, fear of increasing patient anxiety and patients being too ill or too old to be asked.[30, 31]

In the Middle East, the literature is lacking in studies on the proportion of men with diabetes who have been asked about ED or their willingness to discuss ED with their physicians. Also, in searching the literature, no studies were found that described the barriers faced by patients with type 2 diabetes to discuss ED with their physicians.

This study was aimed primarily to find out the proportion of Saudi men with type 2 diabetes who have been asked about ED in the last year by their physicians in hospital-based primary care clinics in Riyadh. We also aimed to determine the willingness of Saudi men with type 2 diabetes to discuss ED with their physicians and the factors that either increase or reduce their willingness to discuss this issue.

## Methods and materials

### Study design

This study employed a cross-sectional survey using a quantitative self-administered questionnaire investigating the proportion of Saudi men with type 2 diabetes who have been asked by their physicians about ED in the last year, their willingness to discuss ED with their physicians, and the factors that may be related to their willingness to discuss ED with their physicians.

### Study site

The study was conducted in hospital-based primary care clinics at King Khalid University Hospital, Riyadh, Saudi Arabia. These primary care clinics consist of 8–10 clinic sessions each day, led by approximately 40 staff specialized in family medicine. Each clinic has about 30 patients daily with the majority of the patients having diabetes.

### Eligibility criteria

The participants were included in this project if they were married, adult (i.e. > 18 years), diagnosed with type 2 diabetes mellitus, having at least one year of follow-up in the clinics, and could read and write Arabic. We excluded any participant with anatomical penile deformities, past history of spinal cord injury or past history of prostate diseases or prostate surgery.

### Patient enrolment

Patients were approached at the reception desk after they completed their review with their physician and asked about the inclusion and exclusion criteria. Those who met our eligibility criteria were included in the study. The participants were informed about the study’s objectives. They were asked to enter the study and for those who accepted this request, written consent was obtained. Confidentiality of their information was assured. The data were collected by a family physician (the principle investigator) from July to September 2015

### Instrument development

The questionnaire consisted of 26 items divided into 4 sections. The first section of the questionnaire collected information about the socio-demographic background of the patients. The second section contained two questions, the first one regarding the proportion of patients with type 2 diabetes who have been asked about ED. The responses were either yes, no, or I can’t remember. The second section measured the degree of willingness to discuss ED by the patients with their physician (i.e. unwilling, slightly willing, moderately willing, and very willing).

The third section consisted of self-reported statements about 11 barriers preventing patients from discussing ED with their physicians. The responses to these statements were recorded on a five point Likert scale ranging from strongly agree to strongly disagree. These statements were developed after reviewing the literature. The last section of the questionnaire comprised a brief survey to assess the presence of ED in respondents by using a validated Arabic translation of the Index of Erectile Function (IIEF-5) questionnaire.[32] IIEF-5 is a well-known tool to screen for ED and it has been used extensively in previous studies. It comprises only five questions.[33] Also, it categorizes ED according to its severity as follow: severe ED (1–7), moderate ED (8–11), mild to moderate ED (12–16), mild ED (17–21), and no ED (22–25).[34]

Apart from the last section of the questionnaire which was adopted from the previously validated Arabic version, the majority of the questionnaire was developed in English, translated by an accredited translator in Arabic, and then back-translated by another accredited translator into English. The mismatches between the two English versions, the original and back-translated versions, were discussed and resolved by the primary author and the translators.

The questionnaire was pretested and piloted on 30 monolingual patients with type 2 diabetes to ensure the comprehensibility and readability of the final Arabic version. The participants in the pilot study were recruited from medical out-patient clinics to prevent contamination with the main sample for the current study.

### Sample size calculation

A sample size calculation was based on a pilot study with 30 participants which showed that 15% of patients with type 2 diabetes had been asked about ED in the last year. So, a sample of 306 patients with type 2 diabetes was required to obtain a 95% confidence interval of +/- 4% around the prevalence estimate of 15%. Assuming 10% of questionnaires in the pilot study were incomplete or not returned, a total of 336 questionnaires was required.

### Statistical analysis

Descriptive statistics were used to describe the study sample characteristics and the participants’ identified barriers to discussing ED with their physicians. To test the association between patients who were willing to discuss (i.e. very willing, moderately willing, and slightly willing) and unwilling to discuss ED with the physicians, and the participants’ socio-demographic and clinical characteristics, chi-square tests were used for categorical variables. In one variable (current occupation), we collapsed some groups together to meet the conditions of the Chi-square test. For continuous variables, the normality of the data was checked by the Kolmogorov-Smirnov test. For normally distributed data, an independent sample t test was used to compare means. For skewed data, the Mann-Whitney U test was used to compare medians.

Chi-square tests were used to compare the association between the willingness to discuss ED with the physician and the different participants’ barriers, after removing the neutral response variable. The Fisher exact test was used to test the association between participants having ED and their willingness to discuss but were not yet asked by their physicians in the last year, as the conditions of the Chi-square test were not met.

Multivariable logistic regression analyses were performed to predict the willingness to discuss ED with the physicians by using the participants’ barriers, socio-demographic and clinical characteristics as covariates. For the logistic regression analysis, participants’ willingness to discuss ED was categorized as a binary variable, comparing those who reported any degree of willingness (very willing, moderately willing, and slightly willing) with those who were unwilling.

The data were analysed using the statistical software package IBM SPSS Statistics for Windows, Version 22.0 (IBM Crop., Armonk, NY, USA).[35] A p-value of less than 0.05 was considered to be statistically significant for all analyses.

### Ethics

The study protocol was reviewed and approved by the Monash University Human Research Ethics Committee and the Institutional Review Board of King Khalid University Hospital, Riyadh, Saudi Arabia, where the data were collected. The participants were informed about the study’s objectives and their permission to enter the study was requested. Written consent was obtained from the participants. Confidentiality of their information was assured.

## Results

### Participants’ socio-demographic and clinical characteristics

Out of the 336 distributed questionnaires, 309 were completed and returned. The response rate was therefore 92%. The median age of the respondents was 60 years and the median duration of diabetes among the respondents was 10 years, with over half (59.2%) on tablets alone as treatment for this condition. Few (9.7%) had been asked by their physicians about ED in the last year although most (84.8%) were willing to discuss this problem with them. The presence of ED among the respondents was 89% with one third of them (28.2%) suffering from severe ED. The remaining socio-demographic and clinical characteristics are shown in Table 1.

**Table 1:** Participants’ socio-demographic and clinical characteristics (N = 309).

Patients’ characteristics		n^a^ (%)
**Age**	Median, (IQR^b^)	60, (10)
**Highest education level**	No school attended	42 (13.6)
	Primary school attended	38 (12.3)
	Secondary school attended	52 (16.8)
	Tertiary school attended	81 (26.2)
	University or college	80 (25.9)
	Master or PhD	16 (5.2)
**Location of residency**	Riyadh	238 (77)
	Outside Riyadh	71 (23)
**Monthly income**	<5000 SR^c^	106 (34.3)
	5000–10000 SR	93 (30.1)
	10001–15000 SR	61 (19.7)
	>15000 SR	49 (15.9)
**Current occupation**	Unemployed	11 (3.6)
	Soldier	1 (0.3)
	Governmental work	89 (28.8)
	Private company	10 (3.2)
	Businessman	27 (8.7)
	Retired	171 (55.3)
**Smoking status**	Never smoked	187 (60.5)
	Current smoker	31 (10.1)
	Former smoker	91 (29.4)
**Duration of diabetes (years)**	Median, (IQR)	10, (12)
**Type of diabetes treatment**	Diet only	8 (2.6)
	Tablets only	183 (59.2)
	Insulin only	37 (12)
	Tablets and insulin	81 (26.2)
**Patients been asked in the last year about ED**	Yes	30 (9.7)
	No	250 (80.9)
	Do not remember	29 (9.4)
**Willingness to discuss ED**	Unwilling	47 (15.2)
	Slightly willing	21 (6.8)
	Moderately willing	53 (17.2)
	Very willing	188 (60.8)
**Presence of ED**	Yes	275 (89)
	No	34 (11)
**Severity of ED**	Mild	54 (17.5)
	Mild to moderate	91 (29.4)
	Moderate	43 (13.9)
	Severe	87 (28.2)

^a^ n = Number

^b^ Interquartile Range

^c^ Saudi Riyal

### Prevalence of identified participants’ barriers to discussing ED with their doctors

Table 2 shows the distribution of participants’ barriers to discussing ED with their physicians. The most prevalent barriers among these respondents were having sex is not important to me (49.5%) and the treatment is too expensive (24.6%).

**Table 2:** Distribution of identified participants’ barriers to discussing ED with their physicians.

Factors that might affect willingness to discuss ED with the physician.	Disagree n^a^ (%)	Neutral n (%)	Agree n (%)
It may embarrass my doctor	225 (72.8)	29 (9.4)	55 (17.8)
My doctor would not take my concerns seriously	170 (55)	81 (26.2)	58 (18.8)
I would feel embarrassed to talk about it	239 (77.3)	26 (8.4)	44 (14.2)
I don’t think there is any effective treatment	197 (63.8)	61 (19.7)	51 (16.5)
I am too sick now to address erectile dysfunction issue.	226 (73.1)	28 (9.1)	55 (17.8)
Having sex is not important to me	129 (41.7)	27 (8.7)	153 (49.5)
The treatment is too expensive	71 (23)	162 (52.4)	76 (24.6)
The treatment will harm my health	114 (36.9)	120 (38.8)	75 (24.3)
I am too old now	219 (70.9)	41 (13.3)	49 (15.9)
It is a personal issue	236 (76.4)	30 (9.7)	43 (13.9)
My doctor is too young to discuss my erectile dysfunction with him	229 (74.1)	35 (11.3)	45 (14.6)

^a^n = Number

### Willingness to discuss erectile dysfunction (ED) and participants’ socio-demographic and clinical characteristics

Table 3 shows the association between the willingness of respondents to discuss ED with their physicians, and the respondents” socio-demographic and clinical characteristics. The participants who were willing to discuss ED with their physicians were younger with the mean age being 59.3 compared to the mean age of 65 in unwilling participants (P< 0.001). Participants with low monthly incomes (i.e. <5000 SR) (53.2%) were unwilling to discuss ED with their physicians (P = 0.03). Also, among participants who have ED, those who were complaining of severe ED (63.1) were unwilling to discuss it with their physicians. There were no significant associations between a willingness to discuss ED with the physicians and the highest education level, location of residency, current occupation, smoking status, duration of diabetes, type of diabetes treatment, and presence of ED.

**Table 3:** Association between willingness to discuss erectile dysfunction (ED) with the physician and participants’ socio-demographic and clinical characteristics.

Patients’ characteristics		Willingness to discuss ED		P value^a^
		Unwilling N^b^ (%)	Willing n (%)	
**Age(years)**	Mean	65	59.3	< 0.001
**Highest education level**	No school attended	10 (21.3)	32 (12.2)	0.45
	Primary school attended	8 (17)	30 (11.5)	
	Secondary school attended	6 (12.8)	46 (17.6)	
	Tertiary school attended	10 (21.3)	71 (27.1)	
	University or college	11 (23.4)	69 (26.3)	
	Master or PhD	2 (4.3)	14 (5.3)	
**Location of residency**	Riyadh	35 (74.5)	203 (77.5)	0.65
	Outside Riyadh	12 (25.5)	59 (22.9)	
**Monthly income**	<5000 SR^c^	25 (53.2)	81 (30.9)	0.03
	5000–10000 SR	11 (23.4)	82 (31.3)	
	10001–15000 SR	7 (14.9)	54 (20.6)	
	>15000 SR	4 (8.5)	45 (17.2)	
**Current occupation**	Unemployed	3 (6.4)	8 (3.1)	0.18
	Businessman	7 (14.9)	30 (11.5)	
	Governmental work	8 (17)	82 (31.3)	
	Retired	29 (61.7)	142 (54.2)	
**Smoking status**	Never smoked	34 (72.3)	153 (58.4)	0.15
	Current smoker	2 (4.3)	29 (11.1)	
	Former smoker	11 (23.4)	80 (30.5)	
**Duration of diabetes (years)**	Mean	14.43	12.19	0.3
**Type of diabetes treatment**	Diet only	1 (2.1)	7 (2.7)	0.13
	Tablets only	34 (72.3)	147 (56.1)	
	Insulin only	6 (12.8)	33 (12.6)	
	Tablets and insulin	6 (12.8)	75 (28.6)	
**Presence of ED**	ED	38 (80.9)	237 (90.5)	0.05
	No ED	9 (19.1)	25 (9.5)	
**Severity of ED**	Mild	4 (10.5)	50 (21.1)	< 0.001
	Mild to Moderate	5 (13.2)	86 (36.3)	
	Moderate	5 (13.2)	38 (16)	
	Severe	24 (63.1)	63 (26.6)	

^a^ The Chi square test was used in the analysis for categorical variables and the Mann-Whitney U test for numerical variables

^b^ n = Number

^c^ Saudi Riyal

**Fig 1:**
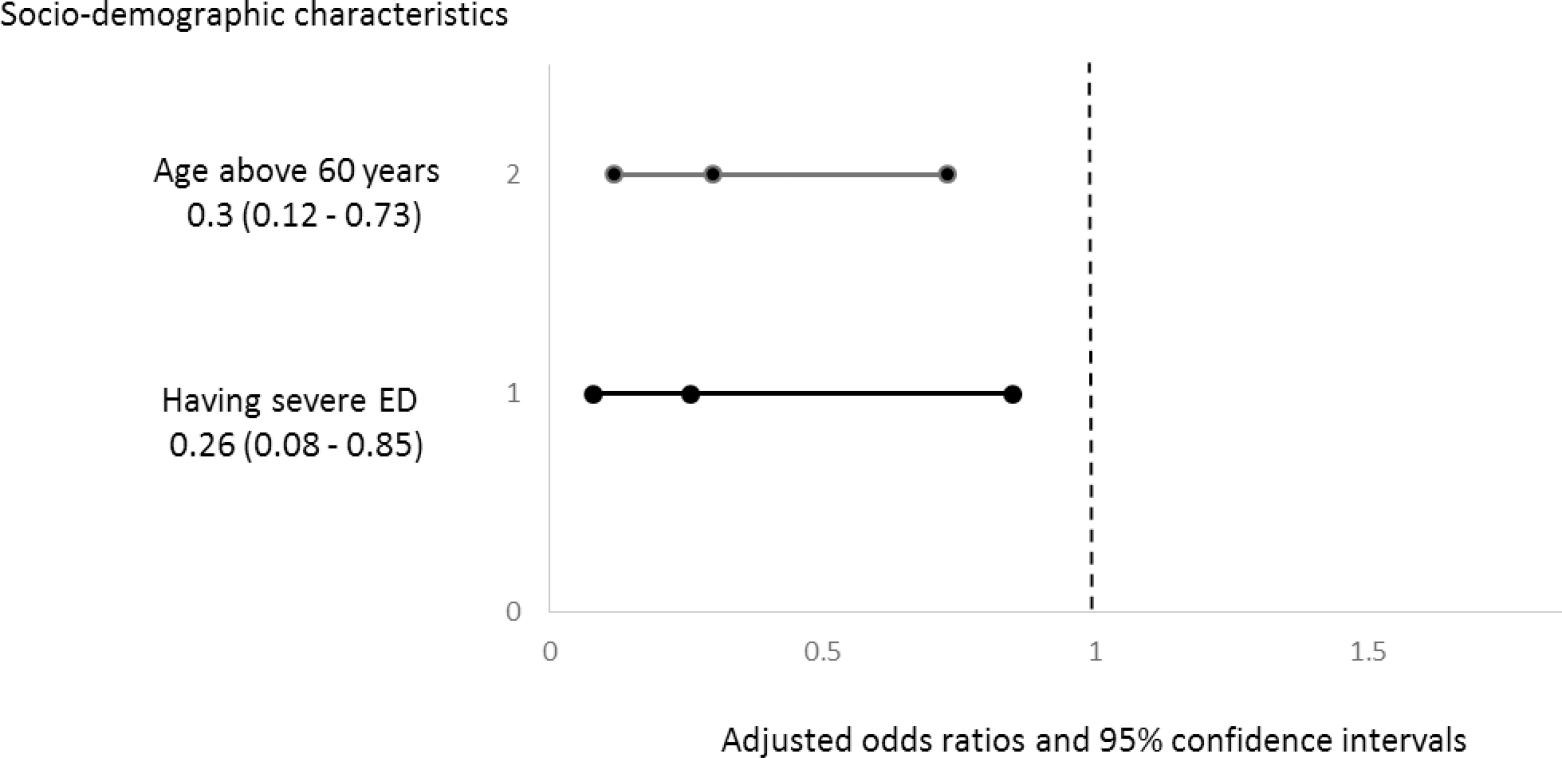
Predicting participants’ willingness to discuss erectile dysfunction (ED).

Multivariable logistic regression analysis was used to predict the participants’ willingness to discuss ED by their socio-demographic and clinical characteristics. After adjusting for the educational level, location of residency, monthly income, current occupation, smoking status, duration of diabetes, type of diabetes treatment, presence of ED, two participants’ characteristics were associated with willingness to discuss ED with the physicians. These characteristics were age above 60 (OR = 0.25, 95% CI: 0.11–0.55), and having severe ED (OR = 0.26, 95% CI: 0.08–0.85) (Fig 1). No significant association has been found between the participants’ willingness to discuss ED and the other socio-demographic and clinical characteristics.

### Participants’ willingness to discuss ED and identified barriers

Table 4 shows the comparison between participants’ willingness to discuss ED with their physicians and the identified barriers. Comparing the ‘unwilling’ participants to the ‘willing’ ones revealed that the barriers which provide the main obstacles to discussing ED with the doctors are: embarrassing my doctor (63.9%, P < 0.001), ED is a personal issue (60.6%, P < 0.001), too old now (59.4%, P < 0.001), feeling embarrassed to talk about it (57.1%, P < 0.001), too sick now to address ED issues (55.9%, P < 0.001), no effective treatment is available (54.8%, P < 0.001), and my doctor is too young to discuss my ED with him (54.8%, P < 0.001).

**Table 4:** Comparison between participants’ willingness to discuss ED with the physicians, and identified barriers.

Patients’ barriers		Patients willingness to discuss		Total n (%)	P^a^ value
		Unwilling n^b^ (%)	Willing n (%)		
**It may embarrass my doctor**	Agree	23 (63.9)	32 (13.1)	55 (19.6)	<0.001
	Disagree	13 (36.1)	212 (86.9)	225 (80.4)	
	Total	36 (100)	244 (100)	280 (100)	
**My doctor would not take my concerns seriously**	Agree	12 (48)	46 (22.7)	58 (25.4)	<0.001
	Disagree	13 (52)	157 (77.3)	170 (74.6)	
	Total	25 (100)	203 (100)	228 (100)	
**I would feel embarrassed to talk about it**	Agree	20 (57.1)	24 (9.7)	44 (15.5)	<0.001
	Disagree	15 (42.9)	224 (90.3)	239 (84.5)	
	Total	35 (100)	248 (100)	283 (100)	
**I don’t think there is any effective treatment**	Agree	17 (54.8)	34 (15.7)	51 (20.6))	<0.001
	Disagree	14 (45.2)	183 (84.3)	197 (79.4)	
	Total	31 (100)	217 (100)	248 (100)	
**I am too sick now to address erectile dysfunction issue**	Agree	19 (55.9)	36 (14.6)	55 (19.6)	<0.001
	Disagree	15 (44.1)	211 (85.4)	226 (80.4)	
	Total	34 (100)	247 (100)	281 (100)	
**Having sex is not important to me**	Agree	130 (52.8)	130 (52.8)	153 (54.3)	0.21
	Disagree	116 (47.2)	116 (47.2)	129 (45.7)	
	Total	246 (100)	246 (100)	282 (100)	
**The treatment is too expensive**	Agree	9 (60)	67 (50.8)	76 (51.7)	0.5
	Disagree	6 (40)	65 (49.2)	71 (48.3)	
	Total	15 (100)	132 (100)	147 (100)	
**The treatment will harm my health**	Agree	11 (55)	64 (37.9)	75 (39.7)	0.13
	Disagree	9 (45)	105 (62.1)	114 (60.3)	
	Total	20 (100)	169 (100)	189 (100)	
**I am too old now**	Agree	19 (59.4)	30 (12.7)	49 (18.3)	<0.001
	Disagree	13 (40.6)	206 (87.3)	219 (81.7)	
	Total	31 (100)	236 (100)	269 (100)	
**It is a personal issue**	Agree	20 (60.6)	23 (9.3)	43 (15.4)	<0.001
	Disagree	13 (39.4)	223 (90.7)	236 (84.6)	
	Total	33 (100)	246 (100)	279 (100)	
**My doctor is too young to discuss my erectile dysfunction with him**	Agree	17 (54.8)	28 (11.5)	45 (16.4)	<0.001
	Disagree	14 (45.2)	215 (88.5)	229 (83.6)	
	Total	31 (100)	243 (100)	274 (100)	

^a^ Chi square test was used in the analysis

^b^ n = ^1^ Number

### Predicting participants’ barriers to their willingness to discuss ED

Table 5 shows the multivariable logistic regression analysis which was used to predict the participants’ willingness to discuss ED by their identified barriers. After adjusting for the age and severity of ED as possible confounders, two participants’ barriers were associated with willingness to discuss ED with the physicians. These barriers were “it may embarrass my doctor” (OR = 0.04, 95% CI: 0.01–0.2), and “It is a personal issue” (OR = 0.05, 95% CI: 0.01–0.28) (Table 5).

**Table 5:** Predicting participants’ barriers to their willingness to discuss ED.

Adjusted by other variables			
Variable	P-Value	OR^a^	95% CI^b^
**It may embarrass my doctor**			
**Disagree (ref**^**c**^**)**	-	-	-
**Agree**	< 0.01	0.04	0.01–0.2
**It is a personal issue**			
**Disagree (ref**^**c**^**)**	-	-	-
**Agree**	< 0.01	0.05	0.01–0.28
**Age (years)**			
**60 or less (ref**^**c**^**)**	-	-	-
**Above 60**	0.02	0.2	0.05–0.80
**ED severity**			
**Mild (ref**^**c**^**)**	-	-	-
**Mild to Moderate**	0.58	1.6	0.29–9.33
**Moderate**	0.17	0.28	0.045–1.72
**Severe**	< 0.01	0.08	0.01–0.49

^a^ Odds ratio.

^b^ Confidence interval

^c^ Reference group

### Participants who have not been asked about ED and their willingness to discuss it

Table 6 shows that among the respondents who have not yet been asked about ED in the last year by their physicians, 91% of them have ED and would be willing to discuss it with their physicians (P = 0.02). Even if they do not have ED, twice as many are willing to discuss this matter as unwilling.

**Table 6:** Association between participants who have not been asked about ED and their willingness to discuss according to whether they have ED.

ED	Have not been asked about ED by their doctor in the last year		P^a^ value
	Unwilling to discuss n^b^ (%)	Willing to discuss n (%)	
**Have ED**	30 (76.9)	192 (91)	0.02
**No ED**	9 (23.1)	19 (9)	

^a^ Fisher exact test was used in the analysis

^b^ n = Number

## Discussion

This survey has shown that few (9.7%) patients with type 2 diabetes mellitus have been asked about ED in the past year by their physicians, in spite of the majority (84.8%) being willing to discuss it. Further, the presence of ED was high (89%) among these patients, with one third of them (28.2%) suffering from severe ED.

In spite of guidelines recommending physicians to enquire about ED in patients with diabetes, this does not take place in most cases.[27,36] Grant et al found that only 9% of the patients with diabetes have been asked about ED in their last diabetes review consultation.[26] In addition, Perttula found that physicians discussed ED with just 18% of their patients who have diabetes.[28] This is in spite of the prevalence of ED being high in these patients.[13,17,37] Also, the low rate of asking about ED in patients who have diabetes by physicians who work in primary care settings is similar to what happens in specialty practices where patients are at high risk for ED, such as those seen by cardiologists. Nicolai et al found that only 16% of cardiologists admitted to discussing sexual function regularly with their patients.[38] So, there is a wide gap between recommendations and what takes place in practice. A study done in Bulgaria has shown that this gap reflects, in part, physicians’ beliefs that patients with ED rarely share this problem with their physicians, [39]

Overall, a large majority of patients (84.8%) were willing to discuss this topic. Unfortunately, to date few studies have examined ED-related issues from the patient perspective. Jiann et al showed that 56.6% of patients with type 2 diabetes wished to discuss ED with their physicians [29] while Lo et al found that 76.1% of patients with type 2 diabetes would want to receive treatment for ED from their physicians.[40] However, most patients think that the discussion should be initiated by the physicians.[29] At the same time, most physicians seem to assume that patients do not like to be asked about sexual problems.[31,41]

The study’s findings suggest that two main factors were associated with a willingness to discuss ED: age and severity of ED. The patients above 60 years were 70% less willing to discuss ED with their physicians compared to the patients less than 60 years old. In addition, the patients who do have severe ED were 75% less willing to discuss ED with their physicians compared to the patients who have mild ED.

As mentioned above, we found that elderly people were less willing to discuss ED with their physicians compared to younger patients in spite of the majority of the elderly population remaining interested in sexual activity. [42] This group needs to be given more attention by their physicians as they have a very high prevalence of ED.[37, 43] In addition to that, ED is often underreported and underdiagnosed in the older male population. [44] It has been shown that physicians are not proactive in discussing and managing the sexual health of elderly people. [45] In a study done by Harding and Manry in the United States among health care providers, it was found that only 28% of the surveyed health care providers would usually asses the sexual health of elderly patients. [46] Also, a negative attitude has been found in a study done to examine American psychologists’ willingness to assess the sexual health of older adults. [47]

In addition to the age of patients with diabetes as a predictor for willingness to discuss ED, the level of ED severity plays a major role, with this study showing that patients who have diabetes with severe ED are less willing to discuss this with their physicians compared to those with mild ED. This is particularly important as the literature suggests that diabetes is associated with more severe forms of ED. [48–49]

Men with diabetes also require more aggressive therapy to treat ED. In a study done by Walsh et al. it was found that men with diabetes were likely to need more aggressive therapy, and most went on to second line therapy (i.e. penile prosthesis surgery) for ED as these patients were less responsive to first line therapy (oral agents). [50] It is important to identify the group who have diabetes and suffer from severe ED to optimise diabetes control and treat the ED as best one can. This should lead to improvement in both their sexual function and depressive symptoms, as shown by the SUBITO-DE study, an Italian multicentre study. [16]

The main barriers contributing to an unwillingness to discuss ED were: embarrassing the doctor, ED is a personal issue, too old, too sick to address ED issues now, no effective treatment available, and the doctor is too young to discuss ED with. Jiann BP et al found that patients’ embarrassment and false beliefs about ED treatment being either ineffective or harmful accounted for three quarters of the reasons why patients with diabetes will not discuss ED with their physicians. [29] Embarrassment was the key factor preventing this discussion according to Rutte et al. [51] as also shown by Gott M and Hichliff S. [31] Other studies have revealed the importance of other barriers including differences in patient characteristics, i.e. their age, lack of knowledge, and difference in their culture. [41,52] These differences in patients’ barriers found by various studies can be explained by difference in customs, traditions, culture, and health systems.

The study findings also suggest that most (91%) of the patients who have not yet been asked about ED in the last year actually have ED and are willing to discuss it. This is contrary to what has been reported by Smith et al. who found that sexually active men are more likely to discuss sex with their physicians. [53] These discrepancies in findings might be related to differences in sociocultural factors including social norms and attitudes.

The current study has several implications for clinical practice. Firstly, ED is a major problem among patients with type 2 diabetes and this is frequently ignored by physicians even though a majority of these patients are willing to discuss this problem. Physicians who are involved in treating these patients should initiate the discussion.[27, 29, 40] Secondly, patients with diabetes who are older and suffer from severe ED are less likely to discuss ED with their physicians. Targeting this sub-group of patients through education and the building of better relationships between physicians and their patients should help. Thirdly, there are multiple barriers that prevent patients with type 2 diabetes discussing ED with their physicians which could be reduced by better patient education and the addressing of psychological factors.

There are several limitations to this study. The sample was taken from one hospital and may therefore not be generalizable. Also, the patients were taken from primary care clinics affiliated to a teaching hospital so that they might have more severe diabetes, and be older than patients in other primary care clinics. In addition to that, and due to the nature of the study design, the results revealed associations and not necessarily causal relationships between a range of factors and willingness to discuss ED. However, we believe that our findings shed an important light on this very sensitive issue among patients with type 2 diabetes. Also, no comparable work has been done in this country, and so it is of importance within this health care system.

## Conclusions

ED is a highly prevalent condition among patients who have type 2 diabetes. Most of these patients are not asked about ED within the last year of attending a clinic, even though most are willing to discuss it with their physicians. Many patients’ barriers to discussing ED have been identified, including being older and suffering from more severe ED, with these patients being less willing to discuss this with their physicians. Further research is needed to explore the barriers which prevent physicians from discussing ED with their patients who have diabetes.

## Supporting information

S1 File (SAV)
